# A164 RISKS OF SKIN CANCERS PRE- AND POST-INFLAMMATORY BOWEL DISEASE DIAGNOSIS

**DOI:** 10.1093/jcag/gwab049.163

**Published:** 2022-02-21

**Authors:** M Narous, Z Nugent, H Singh, C N Bernstein

**Affiliations:** University of Manitoba, Winnipeg, MB, Canada

## Abstract

**Background:**

Inflammatory bowel disease (IBD) has been shown to be associated with an increased risk of non-melanoma skin cancers (NMSC), specifically squamous cell carcinoma (SCC) and basal cell carcinoma (BCC), with the use of immunosuppressants such as thiopurines and tumor necrosis factor inhibitors (anti-TNFa). However, there is no literature exploring risk of skin cancers prior to formal Crohn’s disease (CD) or ulcerative colitis (UC) diagnoses. An increased risk pre IBD diagnosis could potentially suggest varied immunological impairments in patients with IBD.

**Aims:**

To compare risks of NMSC and melanoma preceding and following a diagnosis of IBD and to evaluate the effect of thiopurines and anti-TNFa on risk of skin cancers in IBD.

**Methods:**

This is a retrospective, historical cohort study using population-based data sources including the University of Manitoba IBD Epidemiology Database and the Manitoba Cancer Registry. Individuals with IBD diagnosed between 1987 and 2018 (N = 9344 / 636 NMSC) were identified and matched with randomly selected controls (N = 88916 / 3839 NMSC) based on age, sex, and postal area of residence on the date of IBD diagnosis (index date). Logistic and Cox regression analyses were performed to calculate adjusted risks skin cancers prior to and after IBD diagnosis.

**Results:**

Subjects with IBD were more likely to have BCC pre-dating their IBD diagnosis (OR 1.50, 95% CI 1.24–1.81). Risks of SCC, other non-melanoma skin cancers, or melanoma prior to IBD diagnosis were not significantly increased. Post-IBD diagnosis, risk of BCC and SCC were significantly increased across all IBD groups (HR 1.60, 95% CI 1.45–1.76 and HR 1.65, 95% CI 1.37–1.99), except for SCC in UC. There was no significant association between melanoma and IBD post-IBD diagnosis. Thiopurines are associated with higher risks of BCC, SCC, and melanoma in IBD. Anti-TNFa use also raised risks of BCC and melanoma, but anti-TNFa alone did not increase risk of SCC in IBD. Nested case-control analysis confirmed a higher baseline risk of BCC in patient with IBD with censoring of both thiopurines and anti-TNFa. Similarly, censoring of both medications produced no effect on risk of SCC in IBD corroborating the absence of a baseline SCC risk in IBD.

**Conclusions:**

The risk of BCC preceding a formal diagnosis of IBD is higher than in non-IBD controls, compared to a generally increased risk of NMSC post-IBD diagnosis. IBD is not associated with a significant risk of melanoma, although risk for melanoma is increased with thiopurine or anti-TNFa exposure. Our study suggests a possible inherent immune impairment in patients with IBD that leads to BCC. Thiopurine and anti-TNF therapy increase the risks for skin cancers evident in persons with IBD after their diagnoses.

**Funding Agencies:**

None

